# Extralobar pulmonary sequestration presenting with torsion in an elderly patient: A case report

**DOI:** 10.1016/j.ijscr.2025.111683

**Published:** 2025-07-15

**Authors:** Ryo Maeda, Mayu Inomata, Hiroshi Nakada, Ryusei Yamada

**Affiliations:** a Department of Thoracic and Breast Surgery, Faculty of Medicine, University of Miyazaki, Miyazaki, Japan; b Department of Radiology, Faculty of Medicine, University of Miyazaki, Miyazaki, Japan

**Keywords:** Case report, Surgery, Extralobar pulmonary sequestration, Torsion, Video-assisted thoracic surgery

## Abstract

**Introduction and importance:**

Extralobar pulmonary sequestration with torsion is an exceptionally rare condition, especially in adults, and can present with nonspecific symptoms such as abdominal pain, making diagnosis challenging. Timely recognition is critical, as delayed treatment may lead to infarction and serious complications. This report describes the case of a patient with extralobar pulmonary sequestration who presented with abdominal pain.

**Case presentation:**

An 83-year-old healthy female presented with increasing severe abdominal pain and mild fever that had developed for the past three weeks. Chest and abdominal computed tomography revealed a non-enhancing mass in the right posterior paravertebral area, with mild pleural effusion. Video-assisted thoracoscopic surgery to establish a definitive diagnosis revealed a yellow-whitish ovoid mass with congestion and necrosis, accompanied by bloody pleural effusion. The mass was connected to the mediastinum via a twisted feeding vessel. The final diagnosis was consistent with extralobar pulmonary sequestration with torsion and infarction. The patient's symptoms were relieved immediately after surgery.

**Clinical discussion:**

Extralobar pulmonary sequestration with torsion is rare in adults. Abdominal pain is the hallmark symptom of this condition. The lack of contrast enhancement in the lesion with no visible feeding vascular pedicle or pleural effusion is imaging signs of pulmonary sequestration torsion, and surgical resection is the standard treatment.

**Conclusion:**

This case highlights the importance of considering pulmonary sequestration in the differential diagnosis of unexplained abdominal pain with posterior mediastinal masses, underscoring the value of surgical exploration for diagnosis and treatment.

## Highlights

• Extralobar pulmonary sequestration with torsion in an elderly patient
• Abdominal pain is the hallmark symptom of torsion.
• The lack of contrast enhancement is an imaging sign, along with no visible feeding vascular pedicle or pleural effusion.

## 1 Introduction

Pulmonary sequestration is a relatively rare congenital lung abnormality with an estimated incidence of 0.15–1.7 % [1]. Pulmonary sequestration is divided into intralobar pulmonary sequestration (IPS) and extralobar pulmonary sequestration (EPS) based on the presence or absence of visceral pleura covering the lung parenchyma [2]. EPS is often associated with other congenital anomalies, including congenital diaphragmatic hernia, congenital heart disease, and congenital pulmonary airway malformation. Without these anomalies, asymptomatic cases may remain undetected until discovered incidentally [3]. Symptomatic EPS caused by pedicle torsion is extremely rare [[4], [5], [6], [7], [8], [9], [10], [11], [12], [13], [14], [15], [16], [17], [18], [19], [20]]. Herein, we report a case of EPS diagnosed in an elderly patient secondary to abdominal pain and vomiting caused by torsion. We presented the clinical symptoms, radiological features, and surgical findings of this rare condition in an elderly patient. This study was conducted in accordance with the principles of the Declaration of Helsinki and the SCARE guidelines [21].

## 2 Case presentation

**Fig. 1:**
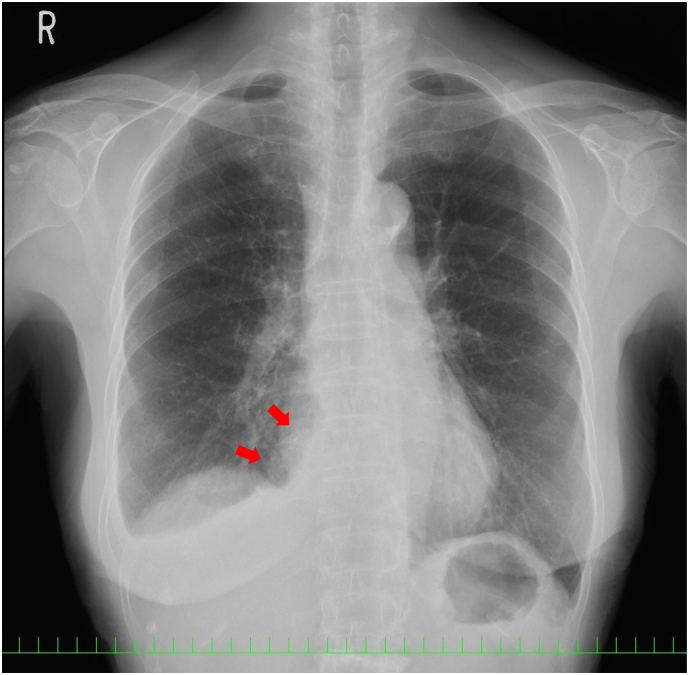
Chest roentgenogram showing a high-density mass (red arrows) in the right thoracic cavity with mild pleural effusion. (For interpretation of the references to colour in this figure legend, the reader is referred to the web version of this article.)

**Fig. 2:**
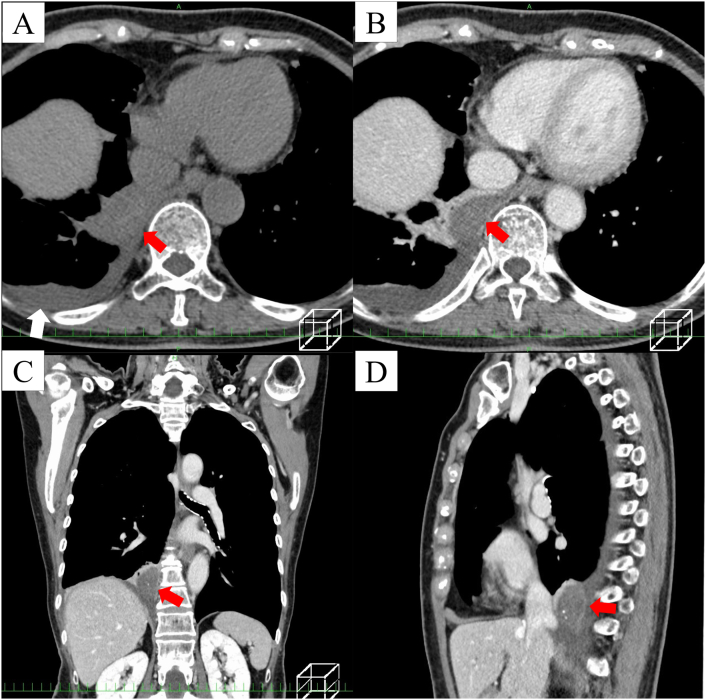
Chest computed tomography (CT) findings. (A) A paraspinal mass is visible in the right posterior mediastinum (red arrow), along with pleural effusion (white arrow). (B) Contrast-enhanced scan showing a well-defined, non-enhancing soft tissue mass in the inferior medial right pleural space, as well as mild left pleural effusion. (C) Chest coronal CT image showing a well-defined soft tissue mass (red arrow) between the diaphragm and the spine in the inferior medial right pleural space without a clear feeding vessel. (D) Chest sagittal CT image showing a mass with slight marginal enhancement (red arrow). (For interpretation of the references to colour in this figure legend, the reader is referred to the web version of this article.)

An 83-year-old healthy female presented at a local hospital with sudden abdominal pain. The physical examination and medical history showed no remarkable findings. The laboratory test results showed a slight elevation in white blood cell count (9510/mL), with 90 % neutrophils. The C-reactive protein level was 0.32 mg/dL, and the other laboratory values were within normal ranges. Chest roentgenography obtained at a local hospital showed a high-density mass in the right thoracic cavity with mild pleural effusion (Fig. 1). Although the patient was treated with oral antibiotics, the increasing symptoms persisted over three weeks. Upper gastrointestinal endoscopic findings were unremarkable. Chest and abdominal computed tomography (CT) scans revealed a well-defined mass with soft tissue density measuring 4.1× 3.9 × 1.8 cm in the right posterior para vertebral area with mild pleural effusion (Fig. 2A–D). The patient was referred to our hospital for further evaluation.

**Fig. 3:**
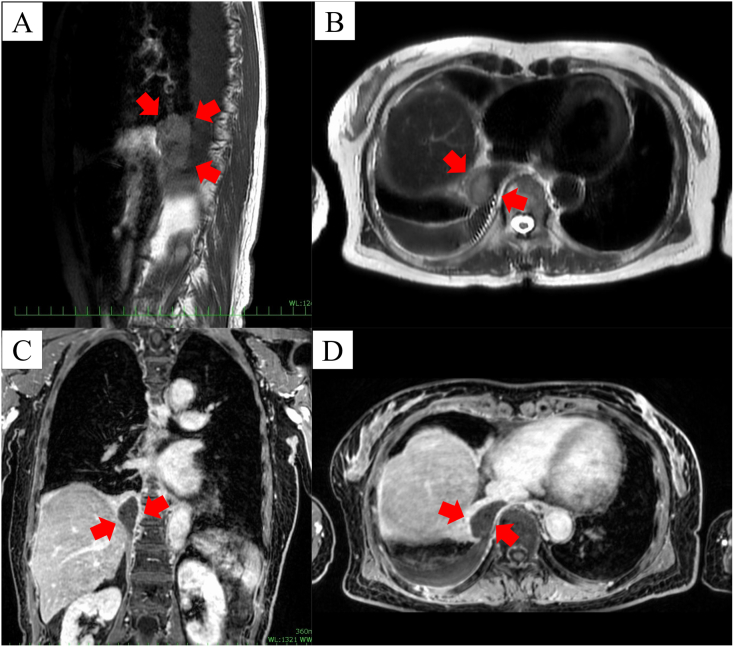
Magnetic resonance imaging findings. T1-weighted sagittal imaging shows a mass with predominantly low signal intensity (red arrows). (C) T2-weighted imaging shows a well-marginated oval mass (red arrows) with mild pleural effusion. (B) Contrast-enhanced T2 weighted coronal image shows a hypointense mass (red arrows) in the inferior medial right pleural space. (D) Contrast-enhanced T2 weighted axial image showing a homogeneous low signal in the mass (red arrows). (For interpretation of the references to colour in this figure legend, the reader is referred to the web version of this article.)

Chest magnetic resonance imaging (MRI) to differentiate pulmonary sequestration with combined torsion from a posterior mediastinal mass revealed a well-marginated mass in the right posterior mediastinum (Fig. 3A). On T2-weighted axial images, the mass showed dark signal intensity without enhancement, a finding suggestive of an old internal hemorrhage and infarction (Fig. 3B–D).

**Fig. 4:**
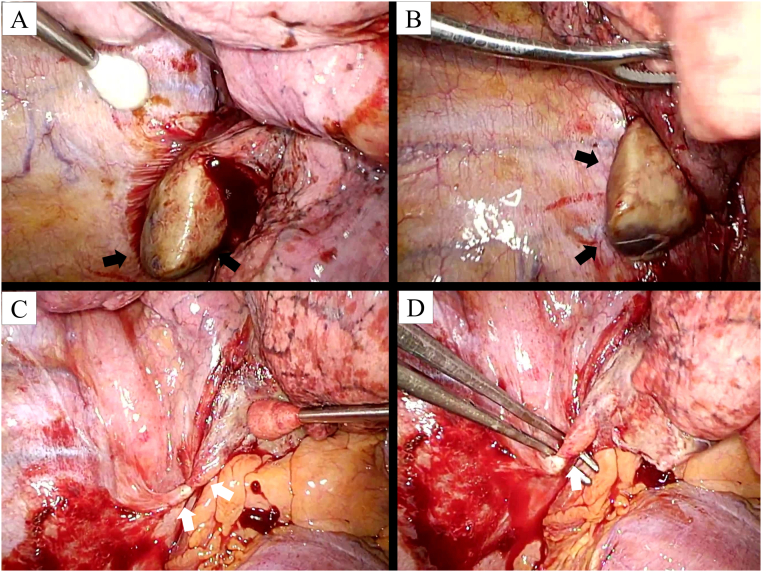
Intraoperative findings. (A) A yellow-whitish mass (black arrows) attached to the chest wall, diaphragm, and right lower lobe is visible. (B) The mass shows congestion and necrosis (black arrows) above the diaphragm, and also displays visceral pleura. (C-D) A twisted feeding vessel connects the mass to the mediastinum (white arrows). (For interpretation of the references to colour in this figure legend, the reader is referred to the web version of this article.)

Three-port video-assisted thoracoscopic surgery was performed to obtain a definitive diagnosis. Two 1.2-cm working ports were inserted into the fifth intercostal space with non-rib spreading, and a 0.5-cm camera port was inserted into the seventh intercostal space at the mid-axillary line. A yellow-whitish ovoid mass with congestion and necrosis was found during surgery, accompanied by bloody pleural effusion (Fig. 4A–B). Inflammation and adhesions were also observed around the lesions (Fig. 4A–B). After isolating the mass from the chest wall, diaphragm, and right lower lobe, it was found to be connected to the mediastinum via a twisted feeding vessel (Fig. 4C–D). This vessel was clamped and transected using a surgical stapler. The mass was then removed with partial resection of the right lower lobe because of severe adhesions. The operative time was 32 min, and blood loss was <10 mL. A chest tube was left after the operation, and it was removed on the day of surgery.

**Fig. 5:**
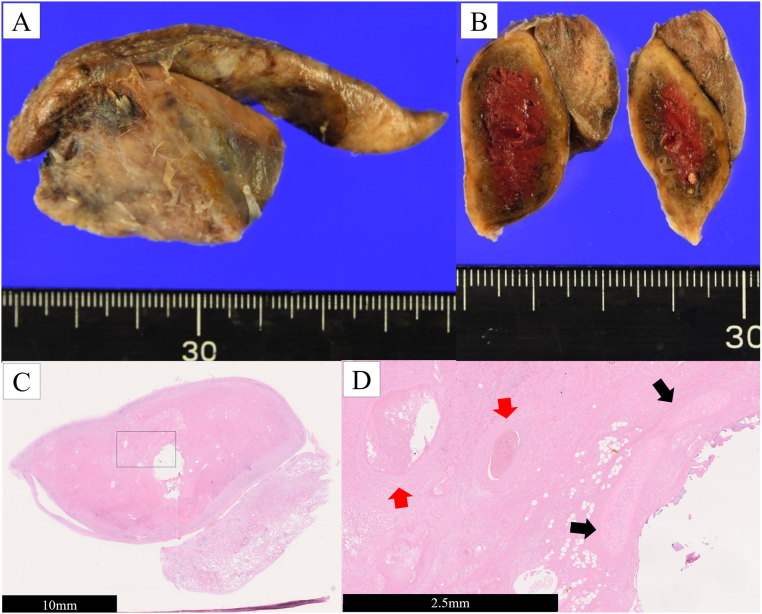
(A) The resected specimen. The mass measured 4.2 × 3.6 × 1.5 cm. (B) A cut section of the excised lesion showing a dark brown hemorrhagic mass. (C) Histological analysis revealed hemorrhagic and necrotic pulmonary tissue (hematoxylin and eosin staining). (D) The inner region of the necrotic tissue. Cartilage tissues (black arrows) and muscular vessels (red arrows) are evident (hematoxylin and eosin staining). (For interpretation of the references to colour in this figure legend, the reader is referred to the web version of this article.)

**Fig. 6:**
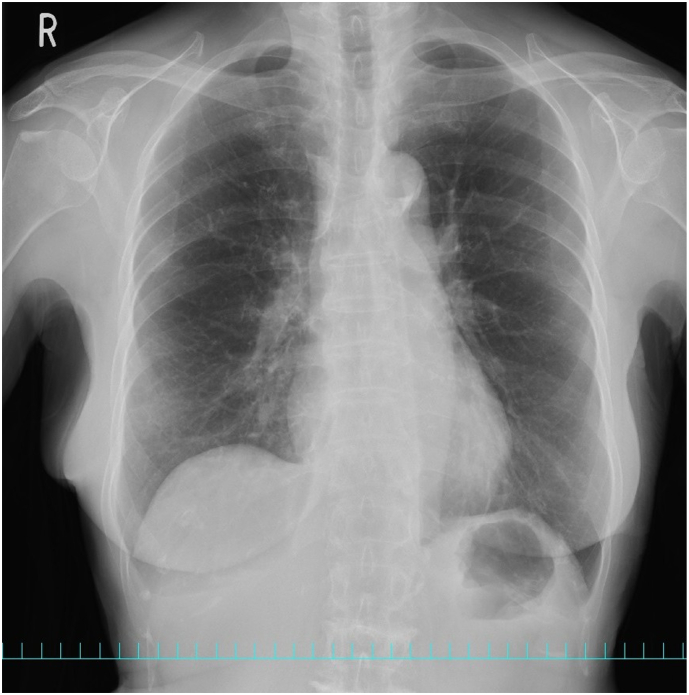
Chest roentgenogram 1 year after the operation showing no apparent tumor shadow, and pleural effusion.

Postoperative pathology revealed infarcted and hemorrhagic lung tissue (Fig. 5A, B). The diagnosis was consistent with EPS with torsion and infarction. The patient's symptoms were relieved immediately after surgery. The patient recovered uneventfully and was discharged on the third postoperative day. No complications or recurrences were observed during the one-year follow-up period (Fig. 6).

## 3 Discussion

Pulmonary sequestration is a rare malformation of nonfunctioning lung tissue that lacks communication with the tracheobronchial tree and receives its arterial blood supply from systemic circulation [22]. IPS, which accounts for approximately 75 % of cases with pulmonary sequestration, is located within a normal lobe and lacks its own visceral pleura, whereas EPS accounts for 25 % of pulmonary sequestrations and is located outside the normal lung [1]. IPS may be complicated with infection, whereas EPS is usually asymptomatic; thus, most cases of EPS are discovered on antenatal ultrasonographic screening or incidentally [10]. Torsion of the vascular pedicle of an EPS is very rare, resulting in infarction, as described in the present case. Unlike IPSs, which are contained within the visceral pleura of the normal lung, EPSs have their own pleural lining and lack the ligamentous attachments of the normal lung. This allows EPSs to rotate on their systemic vascular pedicles, which may result in the strangulation of the sequestration [16]. To date, a total of 25 cases of EPS with torsion have been reported in the English literature [[4], [5], [6], [7], [8], [9], [10], [11], [12], [13], [14], [15], [16], [17], [18], [19], [20]]. EPS with torsion usually presents in children and adolescents [[4], [5], [6], [7], [8], [9], [10], [11],[13], [14], [15], [16], [17], [18], [19], [20]] and cases in adults like in the present case are extremely rare.

Most children present with abdominal pain, vomiting, and flank tenderness, as in our elderly patient [[5], [6], [7], [8], [9], [10],^13^,^15^]. The cause of abdominal pain associated with EPS with torsion could be that the EPS is generally located between the diaphragm and the lower lobe; thus, the pain could be attributed to irritation of the diaphragm caused by the torsion. The exact reasons for torsion remain unclear. The literature suggests that activity or respiratory exertion may be the predisposing factor for vascular pedicle torsion; for example, some patients reported vigorous activity before illness onset [16]. Contrary to literature reporting EPSs in children, our elderly patient did not have a history of similar vigorous activity before symptom onset.

Pulmonary sequestrations always receive an arterial blood supply from the systemic circulation [22]. The presence of a torsed vascular pedicle was confirmed during surgery in our case. As in uncomplicated pulmonary sequestration, the presence of the vascular pedicle in preoperative imaging is a critical clue in diagnosing complicated pulmonary sequestration [23]. In cases with EPS torsion, however, these vessels cannot be visualized due to the lack of blood flow caused by the twisting of the vascular pedicle [19]. As in our case, these aberrant vessels cannot be confirmed in preoperative imaging, including contrast-enhanced CT and MRI. This leads to difficulties in diagnosing torsion in patients with EPS. Among previously published case reports, the correct diagnosis of EPS torsion was suggested preoperatively in a minority of cases [18,20].

Contrast-enhanced CT is the most commonly used technique for diagnosing torsed EPS. The associated pleural effusion in our patient was consistent with most other reports [[4], [5], [6], [7], [8], [9], [10], [11], [12], [13], [14], [15], [16], [17], [18], [19], [20]] and has been attributed to the blockage of the draining lymphatics by torsion of the vascular pedicle. Similar to the case reported by Gawlitza et al. [11], our patient's torsed EPS did not show enhancement following contrast administration. Ti et al. [17] also reported main CT signs of torsed EPS, including lesions with no or mild enhancement, unclear feeding vessels, and rapid increases in pleural effusion or size. The lack of contrast enhancement in the lesion is an imaging sign of pulmonary sequestration torsion, along with no visible feeding vascular pedicle or pleural effusion.

The reported MRI characteristics of EPS with torsion vary. Some cases demonstrate low T2 signal intensity of the torsed EPS [12,14], whereas others show increased or intermediate T2 signal intensities [9,11]. In the present case, the combination of low signal intensity on T2- and T1-weighted imaging with a complete lack of enhancement reflected complete occlusion of the feeding vessel with an advanced degree of infarction of the torsed EPS.

The radiological differential diagnoses in this case included several posterior mediastinal masses such as neurogenic tumors, duplicated cyst, or extramedullary hematopoiesis. Solitary fibrous tumors or other mesenchymal tumors were also considered, but no enhancement indicative of these conditions was observed in the present case.

Surgical resection is the standard treatment for EPS with torsion [[4], [5], [6], [7], [8], [9], [10], [11], [12], [13], [14], [15], [16], [17], [18], [19], [20]]. In recent years, video-assisted thoracic surgery (VATS) is increasingly performed for torsed EPS resection [[14], [15], [16],^18^,^20^]. Owing to difficulties in the preoperative diagnosis of torsed EPS, VATS can provide direct visualization. VATS in the present case revealed that the mass was connected to a vascular pedicle by the visceral pleura. The operative findings and postoperative pathology allowed the definitive diagnosis of torsed EPS in this case.

## 4 Conclusion

Torsion occurs primarily during childhood and adolescence. Although EPS with torsion is extremely rare in adults, clinicians should consider pulmonary sequestration in the differential diagnosis of posterior mediastinal masses with no or only marginal enhancement accompanied by pleural effusion and unclear blood supply arteries. Abdominal pain is the hallmark of sequestration torsion. Surgical resection is the standard treatment for EPS with torsion. Resection using VATS should be performed immediately when EPS with torsion is suspected.

## Abbreviations

IPS: intralobar pulmonary sequestration
EPS: extralobar pulmonary sequestration ()
CT: computed tomography
MRI: magnetic resonance imaging
VATS: video-assisted thoracoscopic surgery

## Author contribution

Dr Ryo Maeda is the writer of this article and corresponding author.

Dr Mayu Inomata, Dr. Hiroshi Nakada and Dr. Ryusei Yamada have reviewed.

## Consent

Written informed consent was obtained from the patient for publication of this case report and accompanying images. A copy of the written consent is available for review by the Editor-in-Chief of this journal on request.

## Ethical approval

As it is a case report, ethical approval is exempted by University of Miyazaki Hospital.

## Guarantor

Dr. Ryo Maeda accepts all responsibility of this article.

## Research registration number

Not applicable.

## Funding

The authors declare that there are no funding sources for their research.

## Conflict of interest statement

All authors have read and approved the final manuscript.
